# Subcellular localization of FOXO3a as a potential biomarker of response to combined treatment with inhibitors of PI3K and autophagy in PIK3CA-mutant cancer cells

**DOI:** 10.18632/oncotarget.14245

**Published:** 2016-12-27

**Authors:** Hyun-Jung Kim, Soo Lee Yoon, Chan Kim Young, Yun Kim Hwan, Woong Ju, Seung Kim Cheol

**Affiliations:** ^1^ Innovative Research Center for Control and Prevention of Women's Cancer, Ewha Womans University Mokdong Hospital, Seoul, Korea; ^2^ Division of Gynecologic Oncology, Department of Obstetrics and Gynecology, Ewha Womans University Mokdong Hospital, School of Medicine, Ewha Womans University, Seoul, Korea; ^3^ Department of Obstetrics and Gynecology, Hanmaeum Changwon Hospital, Changwon, Kyungnam, Korea; ^4^ Department of Obstetrics and Gynecology, The Graduate School, School of Medicine, Ewha Womans University, Seoul, Korea

**Keywords:** PI3K inhibitor, PIK3CA mutant cervical cancer, autophagy inhibitor, FOXO3A, 14-3-3

## Abstract

Autophagy is the process of lysosome-mediated degradation and recycling that functions as an adaptive survival mechanism during anti-cancer therapy. Aberrant activation of the phosphoinositide-3-kinase (PI3K) pathway frequently occurs in solid tumors, including cervical cancer. However, single-agent PI3K inhibitors show modest anti-tumor efficacy in clinics. To see whether autophagy inhibition improves the efficacy of PI3K inhibitor in *PIK3CA*-mutant cancer cells, cells were treated with BKM120, a pan-PI3K inhibitor, and the autophagy inhibitor hydroxychloroquine (HCQ). Autophagy inhibition augmented the efficacy of BKM120 depending on *PIK3CA*-mutant cancer cell type. BKM120 treatment led to the nuclear accumulation of forkhead box O3 (FOXO3a) in Caski and T47D cells, which showed a synergistic effect of BKM120 and HCQ and the strong induction of autophagy. However, most FOXO3a remained in cytoplasm in C33A and ME180 cells, which did not exhibit synergy. These data suggest that BKM120-induced nuclear translocation of FOXO3a might elicit autophagy and be a critical factor determining the synergistic activity of BKM120 and HCQ in *PIK3CA*-mutant cancer cells. The release of FOXO3a from 14-3-3 by BV02 or 14-3-3 knockdown induced autophagy by BKM120 in C33A cells and sensitized the cells to the combined BKM120 and HCQ treatment, suggesting that cytoplasmic retention of FOXO3a by 14-3-3 even in the presence of BKM120 inhibit autophagy induction and synergistic effect of BKM120 and HCQ combination. Taken together, our study shows that subcellular localization of FOXO3a might be a potential biomarker for predicting response to the combination treatment with PI3K and autophagy inhibitors in *PIK3CA*-mutant cervical cancer patients.

## INTRODUCTION

Cervical cancer is the fourth leading cause of cancer-related deaths in women worldwide [1]. Some human papillomaviruses (HPV) are well known to cause this cancer, and the stable insertion of the HPV genome into host DNA is strongly linked to cervical cancer development [2]. Although HPV vaccines have contributed to the prevention of cervical cancers in young women, many patients are still diagnosed at late stages of the disease, when there are limited treatment options and a poor prognosis. Recent advances in targeted therapy against specific somatic mutations have changed the strategies of cancer treatment in general. Somatic mutations including *PIK3CA, PTEN, TP53, STK11*, and *KRAS* are implicated in cervical cancers [3]. Among genes with significantly recurrent somatic mutations in cervical cancer, the mutation frequency of *PIK3CA* encoding the phosphatidylinositol-4,5-bisphosphate 3-kinase catalytic subunit is relatively highly ranked, suggesting that targeted therapy against *PIK3CA* mutations may improve upon current strategies for the treatment of cervical cancer [3, 4].

Preclinical studies and early clinical trials indicate that several phosphoinositide-3-kinase (PI3K) inhibitors demonstrate preferential activity in tumors with *PIK3CA* mutations [5, 6]. However, although long-term stabilization and partial tumor responses have been observed in *PIK3CA*-mutant cancers treated with PI3K inhibitors, the majority of *PIK3CA-* mutant cancers do not show substantial regression in clinical trials. To overcome *de novo* and adaptive resistance to PI3K inhibitors, the underlying mechanisms of drug resistance to PI3K inhibitors and additional therapeutic strategies that increase the efficacy of PI3K inhibitors must be identified.

Autophagy is a highly conserved and tightly regulated cellular catabolic process that involves the lysosomal degradation pathway [7]. Autophagy occurs at basal levels to degrade long-lived cytosolic proteins and organelles in normal physiological conditions, but a large body of evidence indicates that autophagy can also promote tumor cell survival as an adaptive mechanism against cellular stresses, including anti-cancer therapies, depending on the cellular and tissue context [8, 9]. Based on reports that autophagy inhibition can enhance the anti-tumor efficacy of autophagy-inducing therapies, various clinical trials including autophagy inhibitors have been launched [8, 10–12]. To date, the role of autophagy as a potential adaptive mechanism of resistance to PI3K inhibitors has not been investigated in cervical cancer with *PIK3CA* mutations.

Here, we report that autophagy inhibition enhances the anti-tumor efficacy of a PI3K inhibitor in *PIK3CA*-mutant cervical cancer cells depending on the cellular context, in which forkhead box O3 (FOXO3a) nuclear translocation upon treatment with the PI3K inhibitor can induce cytoprotective autophagy, resulting in displaying the synergistic effect of autophagy inhibition with PI3K inhibitor. Our findings indicate that the subcellular localization of FOXO3a might be a potential biomarker for predicting response to the combination treatment with inhibitors of PI3K and autophagy in clinics.

## RESULTS

### Autophagy inhibition enhances the efficacy of a PI3K inhibitor depending on *PIK3CA*-mutant cancer cell type

**Figure 1:**
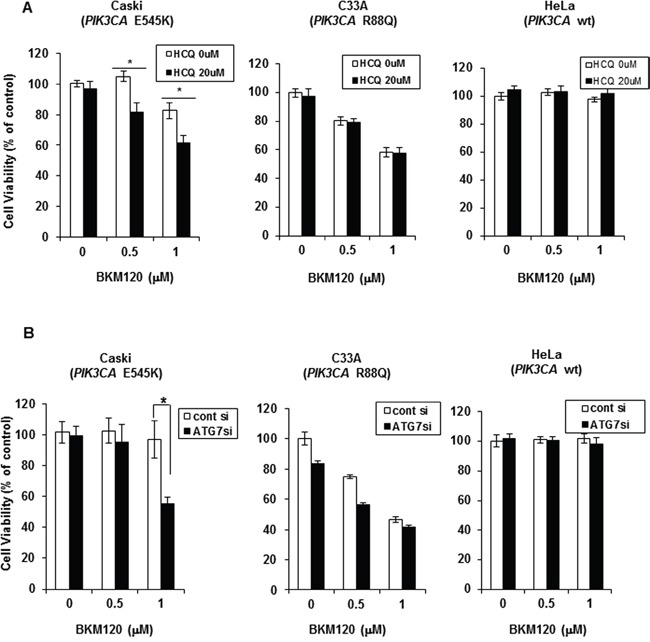
Autophagy inhibition improves PI3K inhibitor efficacy in PIK3CA-mutant cervical cancer cells in a context-dependent manner **A**. Indicated cell lines were seeded in 96-well plates and treated the next day with BKM120 (0.5 and 1 μM) alone or in combination with 20 μM HCQ for 72 h. Cell viability was measured by an MTS-based assay. Columns, means of six replicate determinations; bars ± SD; *P < 0.01. **B**. Indicated cell lines were transiently transfected with ATG7-specific siRNA and then treated with 0.5 μM or 1 μM BKM120 for 72 h. Columns, means of six replicate determinations; bars, ± SD; *P < 0.01.

Although the PI3K pathway is frequently activated in many solid tumors as a result of *PIK3CA* or *AKT* mutations, PI3K inhibitors as single agents are less effective in clinical trials as initially expected [13]. Because autophagy is one of the adaptive mechanisms of resistance to inhibition of the PI3K–AKT pathway [8], we studied whether autophagy inhibition could augment the anti-tumor efficacy of PI3K inhibitor in *PIK3CA*-mutant or wild-type cancer cell lines. *PIK3CA*-mutant cancer cell lines including cervical cancer cell lines (Caski, C33A, and ME-180), breast cancer cell lines (T47D and MCF7), and A2780 ovarian cancer cell line, and wild-type HeLa and SiHa cervical cancer cell lines were treated with BKM120, a pan-isoform PI3K inhibitor that is a leading drug among PI3K inhibitors currently undergoing clinical trials, and hydroxychloroquine (HCQ), an autophagy inhibitor, alone or in combination. *PIK3CA*-mutant cancer cells used in this study have different types of *PIK3CA* mutation; mutations of glutamic acid to lysine at 545 amino acid (E545K) in *PIK3CA* in Caski, ME-180 and MCF7 cells, histidine to arginine at 1047 amino acid (H1047R) in T47D and A2780 cells, and arginine to glutamine at 88 amino acid (R88Q) in C33A. Co-treatment with both drugs resulted in significant synergistic decrease in cell viability in Caski and T47D cells, but no synergism was observed in the other *PIK3CA*-mutant cancer cells (Figure 1A and Supplementary Figure 1). The synergistic effect of BKM120 and HCQ combination in *PIK3CA*-mutant cancer cells is not related to the type of *PIK3CA* mutation and other factors seem to be involved because Caski and MCF7 with the same *PIK3CA* mutation (E545K) showed different responses to the combined treatment of BKM120 and HCQ. *PIK3CA* wild-type HeLa and SiHa did not show significant response to these drugs alone or in combination (Figure 1A and Supplementary Figure 1). To exclude the influence of off-target effects of the drug on the inhibition of autophagy, we treated the cells with small inhibiting (si)RNAs directed against ATG7, which is required for autophagosome formation. Knockdown of ATG7 combined with BKM120 treatment resulted in the significant enhancement of growth inhibition in Caski cells, but not in C33A or HeLa cells (Figure 1B). These results indicate that autophagy inhibition improves the anti-tumor efficacy of BKM120 depending on *PIK3CA*-mutant cancer cell type.

### BKM120 selectively induces autophagy in *PIK3CA*-mutant cancer cell lines

**Figure 2:**
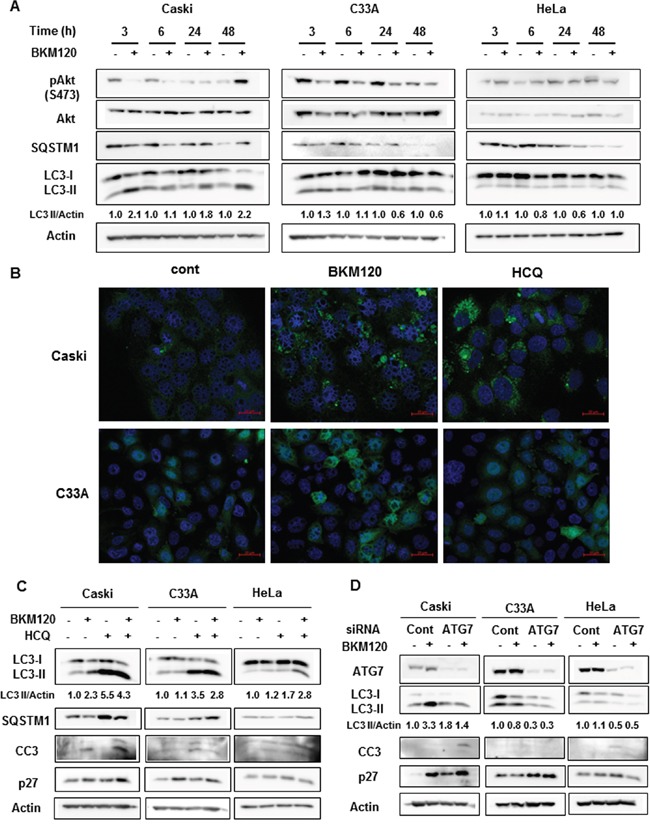
BKM120 selectively increases autophagy in PIK3CA-mutant cancer cells **A**. Cells were treated with 1 μM BKM120 for the indicated periods. Lysate proteins were separated by SDS-PAGE and probed with the indicated antibodies. Actin was used as the loading control. **B**. Stable cell lines overexpressing EGFP-LC3B were treated with 1 μM BKM120 or 20 μM HCQ for 24 h. Representative cells were photographed using a confocal microscope. Scale bar, 20 μm **C**. Cells were treated with 1 μM BKM120 alone or in combination with 20 μM HCQ for 72 h. The lysates were immunoblotted with the indicated antibodies. **D**. Cells were transiently transfected with 20 nM ATG7 siRNA or control siRNA for 24 h and then treated with BKM120 for an additional 72 h. Cell lysates were immunoblotted with the indicated antibodies. LC3-II level was quantified by densitometry and normalized with Actin level. After normalization, LC3-II level was compared to that of non-treated control.

To investigate the role of autophagy in the response to PI3K inhibitors, we assessed autophagy induction in cervical cancer cell lines with or without *PIK3CA* mutations. During autophagy induction, the non-lipidated form of LC3 (LC3-I) is conjugated with phosphatidylethanolamine (PE), then converted into the lipidated form of LC3 (LC3-II), resulting in the increase of LC3-II level or LC3-II/LC3-I ratio [14]. Western blot analysis after BKM120 treatment for the indicated periods revealed a significant increase in the LC3-II level as early as 3 h that was maintained for up to 48 h in Caski cells (Figure 2A), indicating autophagy induction by BKM120 treatment. In contrast, there was no significant increase in LC3-II level upon BKM120 treatment in C33A or HeLa cells. In addition to LC3-II, SQSTM1 has been also examined as a marker of autophagy induction. The SQSTM1 as a cargo protein links LC3 and ubiquitinated substrates, which are degraded during autophagic flux [14]. The decrease in SQSTM1 level was shown at early time points of 3 and 6 hours after BKM120 treatment in Caski cells even though SQSTM1 level did not always inversely correlate with LC3-II level. There was no significant change of SQSTM1 in C33A cells. Unexpectedly, although significant change of LC3-II and Akt phosphorylation levels by BKM120 treatment was not observed in HeLa cells, SQSTM1 level was somewhat affected by BKM120 treatment. It might be explained that some unknown off-targets of BKM120 can affect expression levels of SQSTM1 regardless of PI3K-Akt pathway and autophagy in HeLa cells. To further confirm autophagy induction by BKM120 treatment, we monitored LC3 puncta which indicate autophagosome formation. BKM120 treatment induced LC3 puncta in Caski cells stably expressing EGFP-LC3, but rarely in C33A cells expressing EGFP-LC3 (Figure 2B), indicating that PI3K inhibition by BKM120 induces autophagy in Caski cells but not in C33A even though both cell lines harbor activating mutations in *PIK3CA*. HCQ treatment was here used as a positive control for LC3 puncta (Figure 2B). Due to the different response to BKM120, we examined whether BKM120 effectively blocked the PI3K–Akt signaling pathway in these cell lines. The phosphorylation of Akt at Serine 473 (S473) was effectively inhibited by BKM120 in Caski and C33A cells even though it was not completely blocked (Figure 2A). In Caski cells, the level of Akt phosphorylation at S473 was recovered at the basal level at 24 hours and rather increased compared with the basal level at 48 hours after BKM120 treatment. Considering the result of cell viability assay showing that the treatment of BKM120 alone did not significantly affect the viability of Caski cells (Figure 1), a rapid adaptive resistance mechanism to BKM120 seems to work in these cells. The basal level of Akt phosphorylation at S473 was low and not significantly affected by BKM120 treatment in HeLa cells carrying wild-type *PIK3CA*. These results indicate that PI3K inhibition does not uniformly induce autophagy as a cytoprotective mechanism in all *PIK3CA*-mutant cervical cancer cell.

Next, to investigate whether the blockade of the cytoprotective autophagy induced by PI3K inhibitors enhances apoptosis or cell cycle arrest, the indicated cells were treated with BKM120 alone or in combination with HCQ for 72 h. In correlation with the cell viability assay (Figure 1A), the combination treatment with BKM120 and HCQ increased the levels of the cleaved caspase 3 (CC3) and p27, markers of apoptosis and cell cycle arrest, respectively, compared with single BKM120 or HCQ treatment in Caski cells, but not in C33A or HeLa cells (Figure 2C). Consistent with the data in Figure 2A, BKM120 treatment resulted in increased LC3-II level and decreased SQSTM1 level in Caski cells, clearly indicating autophagy induction; however, C33A and HeLa cells did not show significant changes in LC3-II and SQSTM1 levels upon BKM120 treatment (Figure 2C). HCQ treatment showed significantly increased LC3-II and SQSTM1 levels in Caski and C33A cells, possibly due to autophagosome accumulation resulting from the inhibition of autophagosome and lysosome fusion [15]. Knockdown of ATG7 abrogated the increase of LC3-II induced by BKM120 in Caski cells, resulting in enhanced apoptosis and cell cycle arrest as evidenced by a pronounced increase in CC3 and p27 levels after the combined treatment with BKM120 and siATG7 relative to cells transfected with control siRNA (Figure 2D). Taken together, these data strongly indicate that *PIK3CA*-mutant cervical cancer cells in which BKM120-induced cytoprotective autophagy occurs respond to the combined inhibition of both PI3K and autophagy.

### FOXO3a mediates BKM120-induced cytoprotective autophagy in *PIK3CA*-mutant cervical cancer cells

**Figure 3:**
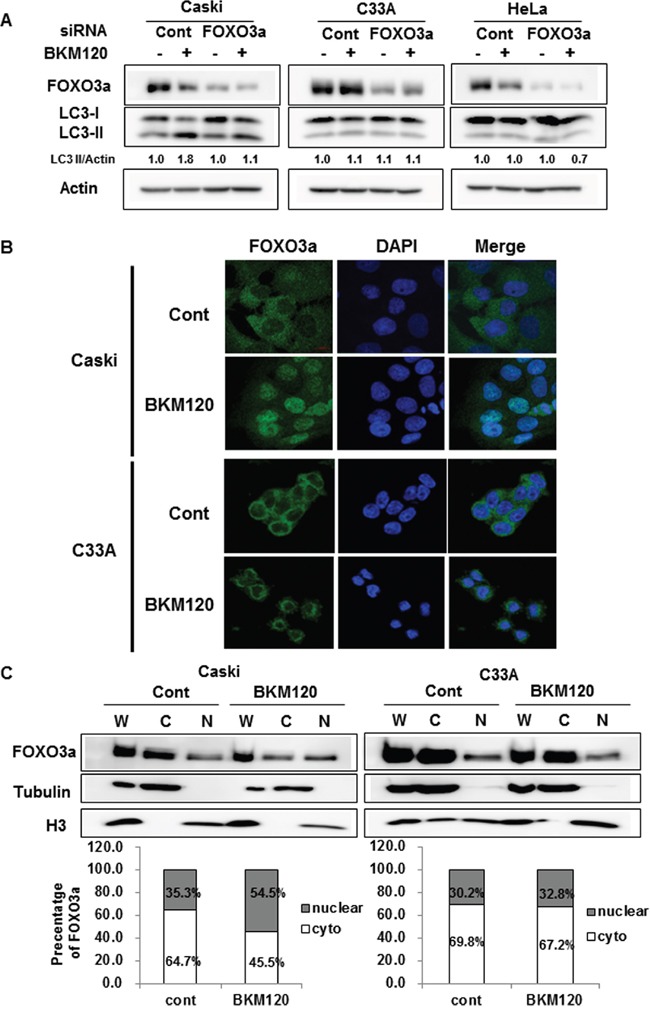
Cellular localization of FOXO3a upon BKM120 treatment plays an important role in autophagy induction in PIK3CA-mutant cancer cells **A**. Cells were transfected with control siRNA or FOXO3a siRNA for 24 h and then treated with 1 μM BKM120 for an additional 24 h. The lysates were immunoblotted with designated antibodies. Actin was used as the loading control. LC3-II level was quantified by densitometry and normalized with Actin level. After normalization, LC3-II level was compared to that of non-treated control. **B**. Cells were cultured on coverslips to approximately 60% confluency and then treated with 1 μM BKM120 for an additional 24 h. Immunofluorescence staining was performed using an anti-FOXO3a antibody and the nuclei were stained with DAPI. Scale bar, 20 μm. **C**. Cells were treated with 1 μM BKM120 for 24 h, followed by lysis of whole-cell extracts and parallel subcellular fractionation. Lysates were immunoblotted with the indicated antibodies. α-tubulin and H3 were used as cytosolic and nuclear markers, respectively. Relative intensity was quantified by densitometry and normalized to the loading controls. The percentage of cytoplasmic and nuclear FOXO3a was determined as follows: cytoplasmic FOXO3a density/(cytoplasmic FOXO3a density + nuclear FOXO3a density) × 100 and nuclear FOXO3a density/(cytoplasmic FOXO3a density + nuclear FOXO3a density) × 100, respectively.

FOXO3a is an important downstream effector of the PI3K–Akt pathway, and the overexpression of FOXO3a in muscle cells induces autophagy by enhancing the expression of autophagy-related genes [16–18]. Activation of the PI3K–Akt pathway sequestrates FOXO3a in the cytoplasm, thus blocking its function as a transcription factor [19]. To examine whether FOXO3a is involved in BKM120-induced autophagy, we knocked down FOXO3a expression using siRNA. Knockdown of endogenous FOXO3a abrogated the BKM120-induced increase in LC3-II level in Caski cells, but not in C33A or HeLa cells (Figure 3A). Knockdown of FOXO3a alone did not affect the basal autophagy. This result strongly indicates that FOXO3a mediates BKM120-induced cytoprotective autophagy in Caski cells.

We wondered why Caski and C33A cells harboring activating mutation in *PIK3CA* respond differently in autophagy induction upon PI3K inhibition. We first examined the cellular localization of FOXO3a in Caski and C33A cells because its subcellular localization is a crucial determinant of its function. Interestingly, confocal immunofluorescence microscopy demonstrated that FOXO3a was translocated from the cytoplasm to the nucleus upon BKM120 treatment in Caski cells, whereas most of FOXO3a was still retained in the cytoplasm in C33A cells (Figure 3B). Cell fractionation analysis further confirmed significant BKM120-induced nuclear translocation of FOXO3a in Caski cells (nuclear FOXO3a levels increased from 35.3% to 54.5%), whereas FOXO3a predominantly remained in the cytoplasmic fraction in BKM120-treated C33A cells (nuclear FOXO3a levels increased from 30.2% to 32.8%) (Figure 3C). To assure whether the difference in subcellular localization of FOXO3a shown in Caski and C33A cells also occurs in other *PIK3CA*-mutant cancer cell lines, subcellular localization of FOXO3a was examined in T47D and ME180 cells. Nuclear translocation of FOXO3a after BKM120 treatment was observed in T47D, which responded to the combined treatment of BKM120 and HCQ (Supplementary Figure 2 and Supplementary Figure 1). In contrast, majority of FOXO3a was still retained in the cytoplasm upon BKM120 treatment in ME180 cells, which did not respond to the combined treatment (Supplementary Figure 2 and Supplementary Figure 1). These results suggest that nuclear translocation of FOXO3a by BKM120 treatment might induce cytoprotective autophagy in Caski and T47D cells and that the cytoplasmic retention of FOXO3a even upon BKM120 treatment might be a major reason why BKM120 did not elicit autophagy in C33A and ME180 cells.

**Figure 4:**
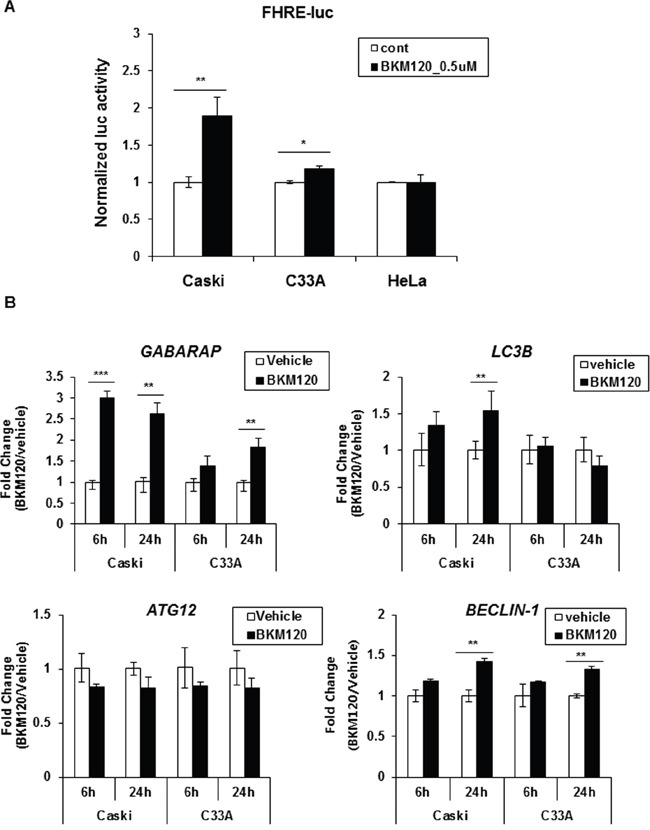
BKM120 induces FOXO transcriptional activity in Caski cells, but not in C33A cells **A**. Cells were transiently co-transfected with FHRE-luc and *Renilla* for 24 h and then treated with 0.5 μM BKM120 for a further 24 h. The cells were harvested and luciferase activity was measured. Firefly luciferase activity was normalized to *Renilla* activity. Data are the mean of three replicates. Bars, ± SD; *P < 0.01, **P < 0.05. **B**. Cells were treated with 1 μM BKM120 for the indicated periods. Total RNA was extracted and mRNA levels of *GABARAP, LC3B, ATG12*, and *Beclin-1* were determined by real-time PCR. The fold-change in mRNA levels was calculated by normalization to *GAPDH*. Data are the mean of three replicates; bars, ± SD; **P < 0.05, ***P < 0.005.

To determine whether BKM120 treatment influences FOXO transcriptional activity, a forkhead-responsive element (FHRE)-luc reporter vector containing three tandem FHREs ligated to a luciferase gene [16] was transiently transfected into three cervical cancer cell lines, followed by BKM120 treatment for 24 h. Promoter activity was significantly increased up to 2-fold upon exposure to BKM120 compared with the control in Caski cells, whereas FOXO activity was slightly or not stimulated by BKM120 in C33A or HeLa cells, respectively (Figure 4A), concordant with the differences in FOXO3a nuclear translocation upon BKM120 treatment. To assure whether the difference in BKM120-induced FOXO activation affects the expression of autophagy-related genes, we examined the gene expression upon BKM120 treatment in Caski and C33A cells. In correlation with the promoter activity shown in Figure 4A, the expression of *GABARAP* and *LC3B* was significantly increased by BKM120 treatment in Caski cells, but only slightly elevated or unaffected in C33A cells (Figure 4B). Unexpectedly, *ATG12* mRNA expression was not stimulated by BKM120 in either cell line, and *BECLIN-1* levels were not significantly different between both cell lines (Figure 4B). Taken together, these results indicate that BKM120 induces the translocation of FOXO3a from the cytoplasm to the nucleus depending on *PIK3CA*-mutant cancer cell type, resulting in the activation of FOXO3a and subsequent autophagy induction.

### Dissociation of FOXO3a and 14-3-3 by BKM120 treatment induces FOXO3a activation and subsequent cytoprotective autophagy in *PIK3CA*-mutant cervical cancer cells

**Figure 5:**
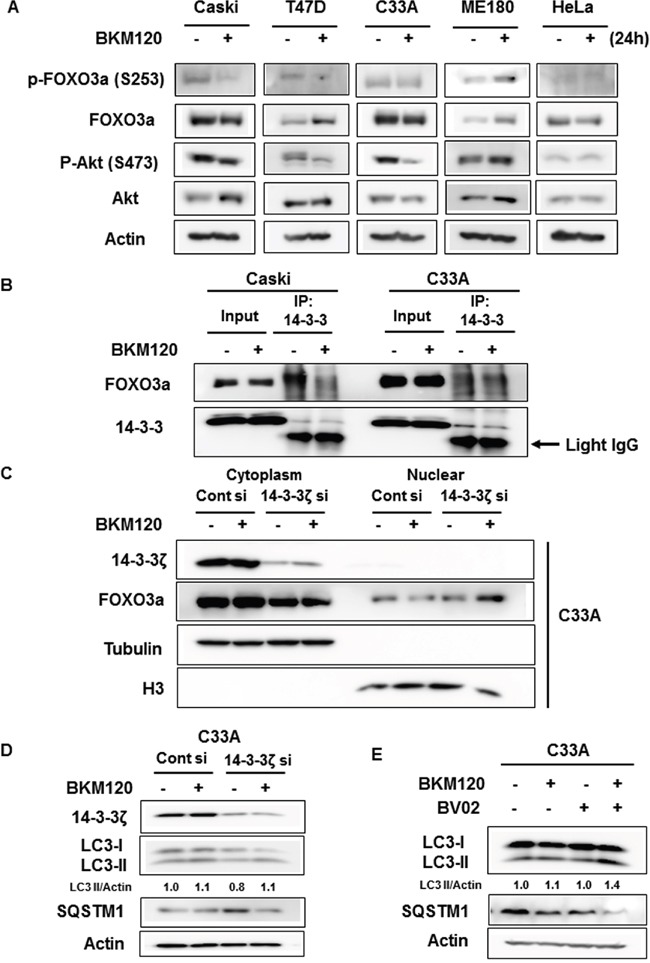
Dissociation of FOXO3a from 14-3-3 by BKM120 treatment mediates FOXO3a activation and autophagy induction **A**. Cells were treated with 0.5 μM BKM120 for 24 h and cell lysates were immunoblotted with the indicated antibodies. Actin was used as the loading control. **B**. Cells were treated with 1 μM BKM120 for 24 h. Cell lysates were immunoprecipitated with anti-pan-14-3-3 antibody, followed by immunoblot analysis for FOXO3a or 14-3-3. Arrow indicates the light IgG chain. **C**. C33A cells were transfected with control siRNA or 14-3-3ζ siRNA for 48 h and then treated with 1 μM BKM120 for 6 h, followed by parallel subcellular fractionation. Lysates were immunoblotted with the indicated antibodies. α-tubulin and H3 were used as cytosolic and nuclear markers, respectively. **D**. C33A cells were transfected with control siRNA or 14-3-3ζ siRNA for 48 h and then treated with 1 μM BKM120 for 6 h. Cell lysates were immunoblotted with the designated antibodies. Actin was used as the loading control. **E**. C33A cells were treated with 1 μM BKM120 alone or in combination with 5 μM BV02 for 6 h. Cell lysates were immunoblotted with anti-LC3B antibody. Actin was used as the loading control. LC3-II level was quantified by densitometry and normalized with Actin level. After normalization, LC3-II level was compared to that of non-treated control.

FOXO proteins are known to be regulated by phosphorylation-dependent nuclear/cytoplasmic shuttling as a result of Akt activity [20]. Akt-mediated phosphorylation of FOXO proteins (at T32, S253, and S315 residues in the case of FOXO3a) induces the binding of 14-3-3 proteins. The resulting complex is translocated to the cytoplasm where the bound 14-3-3 protein prevents re-entry of FOXO proteins into the nucleus, thus negatively regulating their activity [16, 21–23]. To determine whether PI3K inhibition by BKM120 differently affects the phosphorylation of FOXO3a depending on *PIK3CA*-mutant cancer cell type, we examined the phosphorylation status of FOXO3a at the S253 residue because the S253 site is more selective and preferential phosphorylation site by Akt compared with other two Akt phosphorylation sites of FOXO3a and an antibody against phospho-S253 of FOXO3a is commercially available. Interestingly, FOXO3a phosphorylation at S253 was downregulated by BKM120 treatment in Caski and T47D cells, which can cause re-entry of FOXO3a from cytoplasm to nucleus (Figure 5A). However, FOXO3a phosphorylation at S253 was still sustained in C33A cells even though Akt activity was blocked by BKM120 treatment (Figure 5A). In ME-180 cells, FOXO3a phosphorylation seemed to be slightly increased upon BKM120 treatment, attributed by the increase in FOXO3a level and a failure of blocking Akt by BKM120 treatment. These results indicate that the cytoplasmic retention of FOXO3a even in the BKM120 treatment might be due to sustained FOXO3a phosphorylation in C33A and ME-180 cells. The phosphorylation of FOXO3a at S253 was rarely detected and affected by BKM120 treatment in HeLa cells.

14-3-3 proteins are known to interact with Akt-phosphorylated FOXO3a and elicit the export of FOXO3a from the nucleus to the cytoplasm, thus interrupting FOXO3a function. We doubted whether the interaction of FOXO3a with 14-3-3 was sustained even upon the inhibition of PI3K-Akt pathway by BKM120 treatment in C33A cells because Akt-mediated FOXO phosphorylation was not inhibited by BKM120 treatment. We investigated the interaction between FOXO3a and 14-3-3 proteins with or without BKM120 treatment in Caski and C33A cells. As shown in Figure 5B, the association of these proteins was reduced upon BKM120 treatment in Caski cells, but there was no big difference in the interaction between FOXO3a and 14-3-3 with or without BKM120 treatment in C33A cells. We then examined whether disrupting the interaction of FOXO3a and 14-3-3 induces FOXO3a nuclear translocation upon BKM120 treatment in C33A cells. Because 14-3-3ζ was previously reported to bind to FOXO3a upon T32 and S253 phosphorylation of FOXO3a by Akt among 14-3-3 isoforms [16], 14-3-3ζ isoform was knockdowned in C33A cells. The release of FOXO3a from 14-3-3ζ by 14-3-3ζ knockdown induced the nuclear translocation of FOXO3a upon BKM120 treatment in C33A cells (Figure 5C). Moreover, although 14-3-3ζ knockdown decreased basal LC3-II level compared with that of control siRNA, it increased LC3-II level by BKM120 treatment (Figure 5D). Reversely, basal level of SQSTM1 was increased by 14-3-3ζ knockdown relatively to that of control siRNA, but it was significantly decreased by knockdown of 14-3-3ζ combined with BKM120 treatment, indicating that 14-3-3ζ knockdown combined with BKM120 treatment induces autophagy in C33A cells. Consistent with this observation, the combined treatment of BKM120 and BV02, which inhibits the interaction of 14-3-3 proteins with their partners, also induced autophagy in C33A cells as shown by the increase and decrease of LC3-II and SQSTM1, respectively (Figure 5E). Taken together, these findings suggest that the release of FOXO3a from 14-3-3 is required for the induction of autophagy by BKM120 in *PIK3CA*-mutant cancer cells.

### Dissociation of FOXO3a and 14-3-3 sensitizes C33A cells to the combined treatment of BKM120 and HCQ

**Figure 6:**
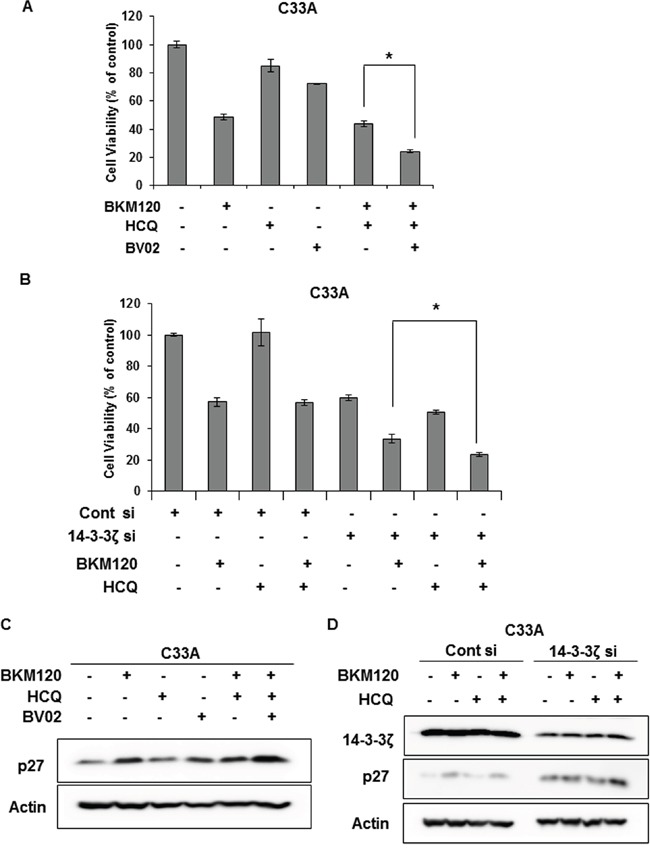
Dissociation of FOXO3a and 14-3-3 sensitizes C33A cells to combination treatment with BKM120 and HCQ **A**. Cells were seeded in 96-well plates overnight and then treated with 1 μM BKM120, 20 μM HCQ, and 5 μM BV02, alone or in combination, for 72 h. Cell viability was measured by the MTS-based assay. Columns, means of six replicate determinations; bars ± SD; *P < 0.01. **B**. Cells were transiently transfected with 14-3-3ζ siRNA and then treated with 1 μM BKM120 alone or in combination with 20 μM HCQ for 72 h. Columns, means of six replicate determinations; bars, ± SD; *P < 0.01. **C**. C33A cells were treated with 1 μM BKM120, 20 μM HCQ, and 5 μM BV02 alone or in combination for 72 h. Cell lysates were immunoblotted with the indicated antibodies. Actin was used as the loading control. **D**. Cells were transiently transfected with 14-3-3ζ siRNA and treated with 1 μM BKM120 alone or in combination with 20 μM HCQ for 72 h. Cell lysates were immunoblotted with the indicated antibodies. Actin was used as the loading control.

To ascertain whether blocking the interaction of FOXO3a and 14-3-3 enhances the efficacy of BKM120 combined with HCQ in C33A cells, we treated cells with BKM120, HCQ, and BV02 alone or in combination for 72 h and then measured cell viability. Compared with BKM120 and HCQ co-treatment, the concurrent treatment of C33A cells with BKM120, HCQ, and BV02 resulted in significantly reduced cell viability (Figure 6A). Additionally, combined BKM120 and HCQ treatment together with 14-3-3ζ knockdown significantly reduced cell viability relative to co-treatment with BKM120 and HCQ in control siRNA-treated cells and treatment with BKM120 alone in 14-3-3ζ knockdown C33A cells (Figure 6B). These results suggest that the release of FOXO3a from 14-3-3 is crucial for the therapeutic response of *PIK3CA*-mutant cancer cells to the combination treatment of PI3K and autophagy inhibitors.

Next, we examined the levels of p27 and CC3 to determine whether the significantly reduced cell viability upon co-treatment with these three drugs in C33A cells is caused by cell cycle arrest or apoptosis. Combined treatment of C33A cells with BKM120, HCQ, and BV02 significantly augmented p27 level compared with treatment with each drug alone or with BKM120 plus HCQ (Figure 6C). Consistently, combined treatment with BKM120 and HCQ together with 14-3-3ζ knockdown also enhanced p27 levels more significantly than BKM120 treatment alone with 14-3-3ζ knockdown or co-treatment with BKM120 and HCQ in control siRNA-treated cells (Figure 6D). However, CC3 or PARP cleavage was not detected under the same conditions (data not shown), indicating that the reduced cell viability by co-treatment with BKM120, HCQ and BV02 or with 14-3-3ζ knockdown might be the result of cell cycle arrest rather than apoptosis.

## DISCUSSION

The use of PI3K inhibitors has resulted in tumor stabilization and shown partial responses in *PIK3CA*-mutant cancers, but dramatic tumor regression has not been commonly seen in clinical trials. Many attempts including combination therapies with other anti-cancer drugs have therefore been doing to improve the modest efficacy of PI3K inhibitors. Autophagy has recently emerged as one of resistance mechanisms against anti-cancer therapy. In this study, we found that autophagy inhibition enhances the efficacy of a PI3K inhibitor depending on *PIK3CA*-mutant cervical cancer cell type. In this context, nuclear translocation of released FOXO3a from 14-3-3 by PI3K inhibitor induces cytoprotective autophagy in *PIK3CA*-mutant cancer cells, leading to the improved anti-cancer efficacy of PI3K inhibitor when combined with autophagy inhibition.

Autophagy occurs at the basal level in virtually all cells as a homeostatic mechanism, but increases when cells face metabolic or therapeutic stresses. In cancer cells, its predominant role is to confer stress tolerance, which maintains tumor cell survival during tumor progression and anti-cancer therapy, leading to drug resistance and disease recurrence [24, 25]. For these reasons, autophagy inhibition combined with anti-cancer therapy has been suggested as a potential therapeutic strategy. In recent studies, the BRAF inhibitor vemurafenib induces cytoprotective autophagy in *BRAF^V600E^* mutant melanoma patients and autophagy inhibition augments BRAF inhibitor-induced cell death and anti-tumor activity both *in vitro* and *in vivo*, and also the concurrent blockade of Akt and autophagy effectively reduces ovarian cancer cell viability [26, 27]. Given the safety and preliminary efficacy of combination therapies with autophagy inhibitors in early clinical studies, the combinatorial strategy of autophagy inhibitors with anti-cancer therapies is quite promising. However, a selection of patients most likely to respond seems to be also needed for improving clinical benefits of these combination therapies. As shown in our study, pharmacologic or RNA interference-based inhibition of autophagy enhances the efficacy of BKM120 depending on *PIK3CA*-mutant cancer cell type. Only *PIK3CA*-mutant cancer cells in which cytoprotective autophagy was induced by BKM120 treatment showed the response to the combined treatment with BKM120 and HCQ, suggesting that autophagy inhibition might display synergism with anti-cancer drugs which induce cytoprotective autophagy, and that other factors such as alterations of protein-protein interaction within cancer cells might affect the induction of cytoprotective autophagy by anti-cancer drugs even in cancer cells with the same gene mutations. Therefore, biomarkers predicting the efficacy of the combined treatment with autophagy inhibitors are required for improved clinical benefit.

This study describes the role of FOXO3a in different responses of *PIK3CA*-mutant cancer cells to the combination of PI3K and autophagy inhibitors and PI3K inhibitor-induced autophagy. It was reported that prolonged FOXO3a activity can cause the resistance to doxorubicin in breast cancer cells and a similar effect was shown in chronic myelogenous leukemia cells [28, 29]. The present study also indicates that FOXO3a might be responsible for the resistance to BKM120 via induction of autophagy in *PIK3CA*-mutant cancer cells. In Caski and T47D cancer cells, FOXO3a was activated and translocated from the cytoplasm to the nucleus upon BKM120 treatment (Figure 3B and Supplementary Figure 2), resulting in the induction of cytoprotective autophagy. However, these events did not occur in C33A and ME180 cancer cells. These findings suggest that autophagy induction via BKM120-induced unclear translocation of FOXO3a determine the cellular response to the combination therapy of PI3K and autophagy inhibitors. Additionally, based on the data from T47D breast cancer cells, it seems that this mechanism might also occur in other *PIK3CA*-mutant cancer types as well as cervical cancer with *PIK3CA* mutations, suggesting that the subcellular localization of FOXO3a upon treatment of PI3K inhibitor might become a biomarker predicting the response to the combination therapy with inhibitors of PI3K and autophagy in *PIK3CA*-mutant cancers. Recently, it was reported that HDAC inhibitors induce autophagy through FOXO1-dependent pathways in human cancer cells [30]. Because our study focused only on FOXO3a which shares several target genes with other members of the FOXO family, the involvement of other FOXO proteins including FOXO1 in PI3K inhibitor-induced cytoprotective autophagy cannot be ruled out. Therefore, further studies are necessary to determine the functions and reciprocal actions of other FOXO proteins in these events.

In addition to Akt, JNK and AMP-activated protein kinase (AMPK) are also known as key factors for nuclear/cytoplasmic shuttling and transcriptional activation of FOXO proteins [19, 20]. However, since activities of these kinases were not affected by BKM120 treatment (data not shown), we ruled out these kinases in the modification of FOXO3a by BKM120 treatment. Although our study do not address the regulation of FOXO3a by other factors, our data indicate that the status of Akt-mediated FOXO3a phosphorylation might be an important factor for its nuclear translocation, autophagy induction, and the therapeutic response to the combined treatment with inhibitors of PI3K and autophagy in *PIK3CA*-mutant cancer cells.

14-3-3 proteins are a family of evolutionarily conserved modulators and are ubiquitously expressed in eukaryotes. Seven mammalian isoforms have been identified: β, γ, ε, η, σ, τ, and ζ. These proteins form heterodimers or homodimers and bind to specific motifs on target proteins in a phosphorylation-dependent manner, altering their subcellular localization, stability, and enzymatic activity [31–33]. Since 14-3-3ζ is highly associated with tumorigenesis and poor prognosis, and its knockdown increases the efficacy of chemotherapeutic agents [34–36], we here showed that dissociation of FOXO3a from 14-3-3, in particular the 14-3-3ζ isoform, is a key factor in BKM120-induced cytoprotective autophagy in *PIK3CA*-mutant cancer cells (Figure 5B, , , and 5E). Furthermore, disruption of the binding of FOXO3a and 14-3-3ζ by 14-3-3ζ knockdown sensitized C33A cells to the combined treatment with BKM120 and HCQ (Figure 6A and 6B). Additionally, 14-3-3ζ knockdown alone significantly reduced the viability of C33A cells (Figure 6B), suggesting that 14-3-3ζ might also be a potential therapeutic target in *PIK3CA*-mutant cervical cancer. The function and involvement of other 14-3-3 isoforms in this regulation should be further studied with regard to enhancing the combinatorial efficacy of inhibitors of PI3K and autophagy.

In conclusion, our findings demonstrate that the subcellular localization of FOXO3a during the treatment with PI3K inhibitors might be a valuable biomarker for selecting patients with *PIK3CA*-mutant cancers who might show clinical benefits upon the combination therapy with inhibitors of PI3K and autophagy. However, larger cohort studies using multiple cancer cell lines with *PIK3CA* mutations and animal models are needed to validate these findings before their application in a clinical setting. Furthermore, our study suggests a possible therapeutic benefit of pharmacologic disruption of the association between FOXO3a and 14-3-3 to sensitize *PIK3CA*-mutant cancers that do not respond to the combined therapy with inhibitors of PI3K and autophagy.

## MATERIALS AND METHODS

### Cell culture and establishment of stable cells

All cell lines used in this study were obtained from the American Type Culture Collection (ATCC, Manassas, VA). Cervical cancer cell lines C33A (ATCC, HTB31), HeLa (ATCC, CCL-2), ME-180 (ATCC, HTB33), and SiHa (ATCC, HTB35), and the breast cancer cell line T47D (ATCC, HTB133) were maintained in RPMI-1640 (Hyclone, SH30027) supplemented with 10% fetal bovine serum (FBS) (Gibco, 12483-020) in a 5% CO_2_ atmosphere at 37°C. The cervical cancer cell line Caski was maintained in DMEM (Hyclone, SH30022) containing 10% FBS in a 5% CO_2_ atmosphere at 37°C. Stable Caski and C33A cell lines overexpressing enhanced green fluorescent protein (EGFP)-LC3B were generated by transfection with the EGFP-LC3B expression vector followed by selection using 1 mg/ml G418 sulfate (BioSesang, G1002).

### Reagents, antibodies, and plasmids

BKM120 (S2247) and hydroxychloroquine (S4430) were purchased from Selleck Chemicals. BV02 (SML0140) was purchased from Sigma-Aldrich. Antibodies against the following proteins were used in this study: phospho-Akt S473 (Santa Cruz Biotechnology, sc-7985-R), Akt (Santa Cruz Biotechnology, sc-8312), α-tubulin (Santa Cruz Biotechnology, sc-8035), pan-14-3-3 (Santa Cruz Biotechnology, sc-629), 14-3-3ζ (Santa Cruz Biotechnology, sc-1019), p27 (Santa Cruz Biotechnology, sc-1641), β-actin (Santa Cruz Biotechnology, sc-47778), cleaved caspase-3 (Cell Signaling Technology, #9661), phospho-FOXO3a S253 (Cell Signaling Technology, #13129), FOXO3a (Cell Signaling Technology, #2497), LC3B (Abcam, ab48394), ATG7 (MBL, PM039), and di-methyl histone H3 (Lys9) (Cell Signaling Technology, #9753). The LC3B-EGFP expression vector (#11546) and the FHRE-luc reporter vector (#1789) were from Addgene (Cambridge, MA). The siRNAs used in the present study included ATG7 siRNA (Ambion, Silencer® Select Pre-designed siRNA, Cat#s20651), 14-3-3ζ siRNA (Invitrogen, Stealth siRNA™, cat#5480996), FOXO3a siRNA (Invitrogen, Stealth siRNA™, Cat#5436311), and negative control siRNA (Bioneer, SN-1022).

### Western blot analysis

Protein lysates were prepared using a PRO-PREP protein extraction solution (iNtRON, Cat#17081) according to the manufacturer's protocol. Equal amounts of protein were resolved by sodium dodecyl sulfate polyacrylamide gel electrophoresis (SDS-PAGE) and transferred onto a polyvinylidene fluoride (PVDF) membrane. After blocking with 5% nonfat skim milk, the membrane was blotted with designated primary and secondary antibodies, developed using the enhanced chemiluminescence method (Clarity™ Western ECL Substrate, #170-5060, BIO-RAD), and visualized with ImageQuant LAS4000 mini (GE Healthcare). β-actin was used as the protein loading control.

### Cell viability assay

Cells were seeded at 3,000 to 5,000 cells/well in 96-well plates, depending on the optimal conditions for each cell line. siRNA-transfected cells were plated 24 h after knockdown. Cells were treated with the drugs alone or in combination for 72 h. Viable cells were measured using an MTS-based assay with CellTiter 96 Aqueous One Solution Reagent (Promega, G3580) following the manufacturer's protocol. The absorbance of each well was measured on an ELISA reader VERSA MAX (Molecular Devices) at 490 nm. The proportion of viable cells per treatment group was normalized to that of control wells.

### RNA preparation and real-time PCR

Total RNA was isolated using the RNeasy Mini Kit (Qiagen, #74104). Total RNA (1 μg) was reverse-transcribed into cDNA using the GoScript™ Reverse Transcription System (Promega, A5000) and real-time quantitative PCR (qPCR) was performed using the Power SYBR Green PCR Master Mix (Applied Biosystems, #4367659) on a 7000 real-time cycler (Applied Biosystems). The following thermal conditions were used for real-time PCR: 95°C for 10 min followed by 40 cycles of 95°C for 15 s and 60°C for 1 min. The following primers were used: *GAPDH* forward, 5′-TTCGACAGTCAGCCGCATCTTCTT-3′, reverse 5′-GCCCAATACGACCAAATCCGTTGA-3′; *GABARAP* forward 5′-GGAGAAAAGATCCGGAAGAAA-3′, reverse 5′-TGGCCAACAGTAAGGTCAGA-3′; *LC3B* forward 5′-CGATACAAGGGTGAGAAGCAG-3′, reverse 5′-TTGAGCTGTAAGCGCCTTCTA-3′; *ATG12* forward 5′-CTGGCGACACCAAGAAAAA-3′, reverse 5′-ATGAGTCCTTGGATGGTTCG-3′; *Beclin-1* forward, 5′-GGCTGAGAGACTGGATCAGG-3′, and reverse 5′-CTGCGTCTGGGCATAACG-3′.

### Subcellular fractionation

Cells were collected in phosphate-buffered saline (PBS), and the cell pellet was divided equally between two tubes for whole-cell lysate and cell fractionation. Whole-cell lysates were prepared with 100 μl of lysis buffer containing 25 mM Tris-HCl pH 7.4, 150 mM NaCl, 1% NP-40, 1 mM EDTA, 5% glycerol, protease inhibitor cocktail (Roche, #11-836-153-001), and phosphatase inhibitor cocktail 2 and 3 (Sigma-Aldrich, P5726 and P0044). For cytoplasmic fractionation, 100 μl of cytosol extraction buffer (10 mM Tris-Cl (pH 8.0), 60 mM KCl, 1 mM EDTA, 1 mM DTT, protease inhibitor cocktail, and phosphatase inhibitors) was added and the cells were incubated on ice for 5 min to allow swelling before the addition of 0.5% NP-40, gentle vortexing, and incubation on ice for 5 min. The cells were centrifuged at 4,000 rpm for 5 min at 4°C. The supernatant (cytoplasmic fraction) was transferred into a new tube and the pellets were lysed for nuclear fractionation with 100 μl of nuclear extraction buffer (20 mM Tris-Cl (pH 8.0), 0.4 M NaCl, 1.5 mM MgCl2, 1.5 mM EDTA, 1 mM DTT, protease inhibitor cocktail, and phosphatase inhibitors) containing 0.5% NP-40. After vigorous vortexing, the solutions were incubated on ice for 10 min followed by centrifugation at 14,000 rpm for 10 min at 4°C. The supernatant was harvested as the nuclear extract. Whole-cell lysates, cytoplasmic extracts, and nuclear extracts were resolved by SDS-PAGE, transferred to a PVDF membrane, and the membranes were probed with the designated primary and secondary antibodies. Tubulin and Di-Methyl Histone H3 (Lys9) were used as cytoplasmic and nuclear markers, respectively.

### Co-immunoprecipitation

Lysates were prepared with lysis buffer containing 25 mM Tris-HCl pH 7.4, 150 mM NaCl, 1% NP-40, 1 mM EDTA, 5% glycerol, protease inhibitor cocktail, and phosphatase inhibitor cocktail. The lysates (500 μg) were pre-cleared by incubation with protein A/G agarose beads (Santa Cruz Biotechnology, sc-2003) for 1 h and centrifugation. The supernatant was incubated with 1 μg of pan 14-3-3 antibody overnight with rotation at 4°C. For immunoprecipitation, 20–30 μl of protein A/G agarose beads was added and incubated for 3 h with rotation at 4°C. After centrifugation, the supernatant was discarded and the beads were collected and washed three times with lysis buffer. The bound proteins were eluted by boiling for 5 min in 50 μl 1× SDS-PAGE sample buffer. The immunoprecipitated proteins and the original lysates (input) were resolved by SDS-PAGE, transferred to a PVDF membrane, and probed for FOXO3a or 14-3-3.

### siRNA transfection

Cells were cultured to approximately 70% confluency and transfected with 20–30 nM siRNA using Lipofectamine RNAiMax reagent (Invitrogen, #13778) according to the manufacturer's instructions.

### Reporter transfection and luciferase assay

Cells were cultured in 6-well plates to approximately 70% confluency. FHRE-luc and *Renilla* plasmids were then co-transfected into the designated cells using Lipofectamine 2000 (Invitrogen, #11668) according to the manufacturer's protocol. The next day, cells were treated with BKM120 for 24 h. Luciferase activity was measured using a Dual-Luciferase Reporter Assay System (Promega, E1910) based on the protocol provided by the manufacturer. Firefly luciferase activity in each sample was normalized against *Renilla* luciferase activity.

### Immunofluorescence staining and confocal microscopy

Cells grown on coverslips were fixed in 4% paraformaldehyde, blocked, and incubated with primary and corresponding secondary antibodies (Alexa Fluor 488 conjugated) (Invitrogen, A11034). Mounting medium containing 4′,6-diamidino-2-phenylindole (DAPI) was used to visualize the nucleus (ImmunoBioScience Corp., AR-6501-01). Stable EGFP-LC3B-expressing cells were seeded on coverslips and, after the designated treatment, were fixed with 4% paraformaldehyde followed by permeabilization with PBS containing 0.25% Triton X-100 for 10 min at 4°C. The cells were examined using a confocal microscope (LSM 800, Carl Zeiss) and representative cells were selected and imaged.

### Statistics

Data of the cell viability assay were presented as mean ± SD from six replicates and were analyzed by a two-tailed Student's *t*-test. Differences were considered significant at a value of *P* ≤ 0.05.

## SUPPLEMENTARY MATERIALS AND METHODS AND FIGURES



## Abbreviations

PI3K: phosphoinositide-3-kinase
PIK3CA: phosphatidylinositol-4,5-bisphosphate 3-kinase catalytic subunit
HCQ: hydroxychloroquine
FOXO: forkhead box O
