# Supplementary material for: Subcellular localization of FOXO3a as a potential biomarker of response to combined treatment with inhibitors of PI3K and autophagy in PIK3CA-mutant cancer cells
Source: Oncotarget. 2016 Dec 27;8(4):6608–22. doi: 10.18632/oncotarget.14245 (PMC5351656; doi:10.18632/oncotarget.14245)
Supplement: Supplementary file 1 [file oncotarget-08-6608-s001.pdf]

Subcellular localization of FOXO3a as a potential biomarker of response to combined treatment with inhibitors of PI3K and autophagy in *PIK3CA*-mutant cancer cells

SUPPLEMENTARY MATERIALS AND METHODS

Cell viability assay

Cells were seeded at 3,000 to 5,000 cells, dependent on optimal conditions per cell line, in 96-well plates. siRNA transfected cells were plated 24 hours after knockdown. Cells were treated with drugs alone or in combination for 72 hours. Viable cells were measured using the MTS assay with CellTiter 96 Aqueous One Solution Reagent (G3580, Promega) following the manufacturer's protocol. The absorbance was measured with ELISA reader VERSA MAX (Molecular Devices) at 490nm. The proportion of cells per treatment group was normalized to control wells.

Immunofluorescence staining and confocal microscopy

Cells on coverslips were fixed with 4% paraformaldehyde solution and then cells were fixed with 4% paraformaldehyde solution followed by permeabilization with PBS containing 0.25% Triton X-100 for 10 minutes at 4°C. Cells were blocked with 1% BSA in PBS containing 0.05% Tween 20 and incubated with primary and corresponding secondary antibodies (Alexa Fluor 488 conjugated) (Invitrogen, A11034). Mounting medium containing DAPI (4',6-diamidino-2-phenylindole) was used to visualize the nucleus (ImmunoBioScience Corp., AR-6501-01). Cells were examined and recorded using a confocal microscope (LSM 800, Carl Zeiss) and representative cells were selected and photographed.

SUPPLEMENTARY FIGURES

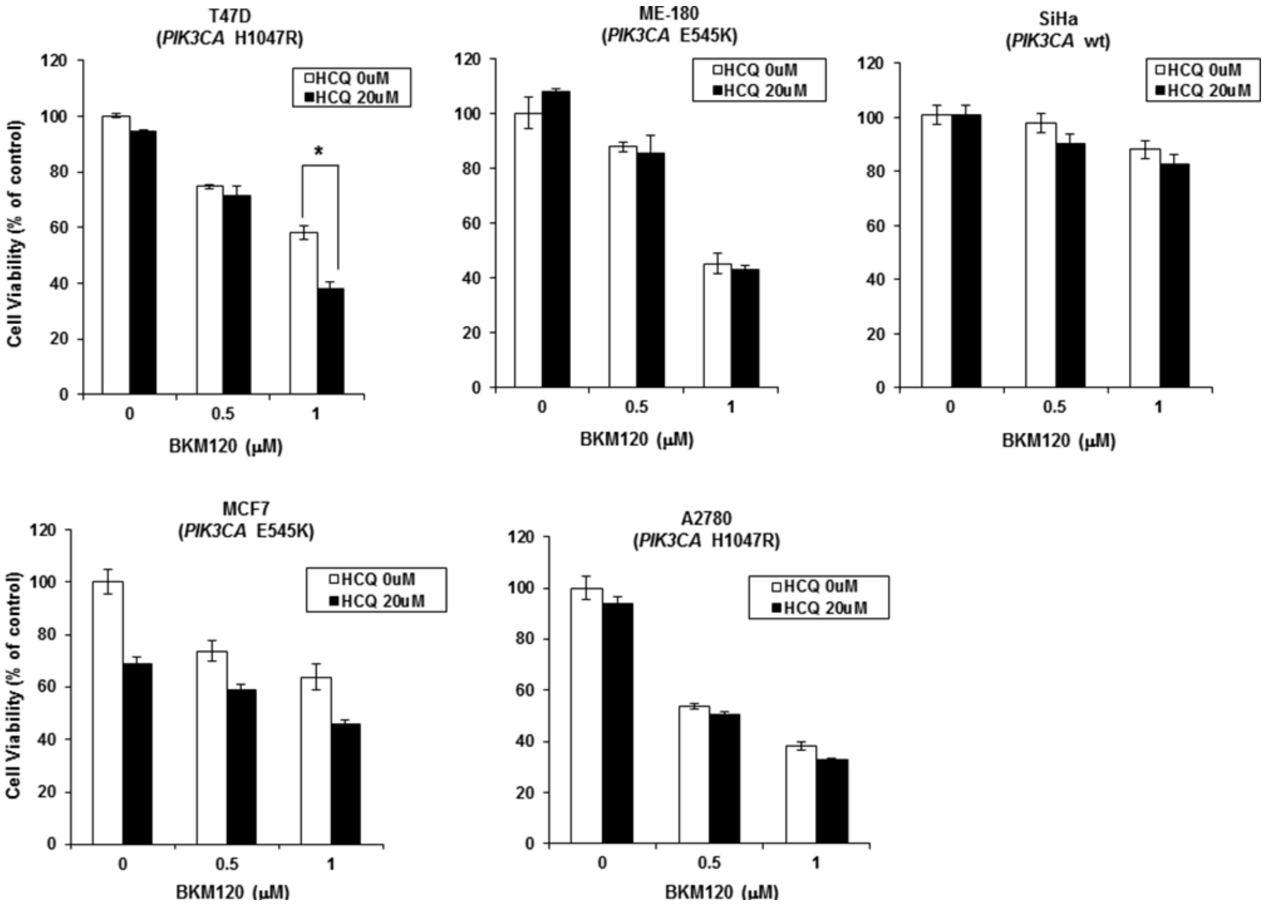

Supplementary Figure 1: Autophagy inhibition improves the efficacy of PI3K inhibitor context-dependently in *PIK3CA* mutant cancer cells. The indicated cell lines were seeded in 96-well plates and treated the next day with BKM120 (0.5 and 1 µM) alone or in combination with 20 µM HCQ for 72 hours. Cell viability was measured by MTS-based assay. Columns, means of six replicate determinations; bars, ±SD. *$P < 0.01$

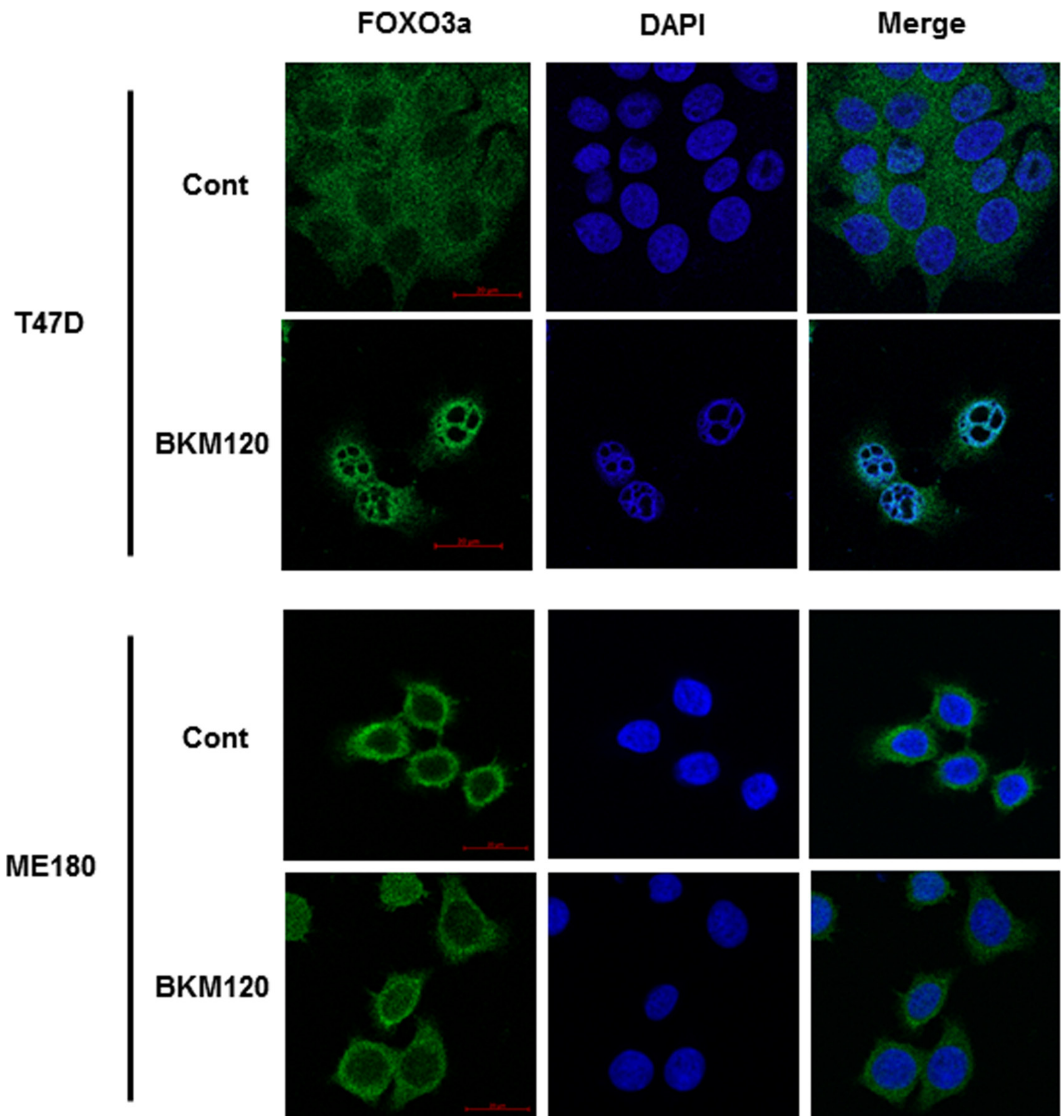

Supplementary Figure 2: Cellular localization of FOXO3a by BKM120 treatment. Cells were cultured on coverslip to reach about 60-70 % confluency followed by treating 1 μM BKM120 for additional 24 hours. Immunofluorescence was performed using anti-FOXO3a antibody and nuclei were stained with DAPI. Scale bar, 20 μM.
